# Beyond Vicarious Storytelling: How Level Telling Fields Could Help Create a Fair Narrative on Migration

**DOI:** 10.12688/openreseurope.15434.2

**Published:** 2024-08-20

**Authors:** Carolin Gebauer, Roy Sommer

**Affiliations:** 1 School of Humanities - English and American Studies, University of Wuppertal, Wuppertal, 42119, Germany

**Keywords:** migration, narrative, vicarious storytelling, level telling field (LTF)

## Abstract

Life stories play a crucial role in migration discourses: they serve as testimony in journalistic work, form the core of ambassadorial storytelling by non-governmental organizations (NGOs), and inspire collaborative projects initiated by writers seeking to express their solidarity. However, this article argues, drawing on migrants’ experiences for such purposes also creates an ethical dilemma: speaking *about*–or even *for*–rather than *with* migrants assigns them a passive role and tends to recycle existing narrative patterns and templates. Starting with a generic distinction between what we call stories of migration (various forms of self-expression granting migrants full authority and control over their narrative) and narratives on migration (external perspectives, *e.g.*, academic, economic, political, and legal approaches, detached from lived experience), we explore the extensive middle ground of hybrid forms between these two extremes– *i.e.*, different kinds of vicarious storytelling–before addressing their ethical implications. We further discuss how the metaphor of the level playing field, a key concept in economics, can be used in transdisciplinary research projects to establish level telling fields (LTFs), *i.e.*, communicative spaces characterized by a fair dialogue on an equal footing for all participants.

## Introduction

In public discourse, migration is often discussed in figurative terms, and especially through the use of inundation metaphors such as waves, floods, or tides. Empirical research on metaphorical representations of migrants and immigration on social media in the United States of America suggests that a “positive association between use of the inundation metaphor and border wall support is present in everyday communication” ( Jimenez *et al*., 2021, p. 167). What is more, inundation metaphors invariably represent individual refugees and migrants as part of an allegedly homogenous group, denying individuals the recognition and respect they deserve. Metaphorical language of this kind neither accounts for lived experiences, including suffering, loss, and trauma, nor does it sufficiently acknowledge that basic human rights are at stake when borders are ‘protected’ by illegal pushbacks or when rescue missions are denied entry to European ports. In addition, inundation metaphors fuel hostility against refugees and migrants, making successful integration, let alone an inclusive welcome culture, difficult if not impossible to achieve. Even worse, metaphors like “refugee waves” as well as derogatory expressions such as “mass immigration” tap into the “Great Replacement conspiracy theory” ( Butter & Knight, 2020, p. 2), an Islamophobic narrative claiming that Europe’s cultural identity is threatened by liberal immigration policies. ^1^

One way of challenging such stereotypical representations is a humanitarian approach based on narratives and stories. Our argument starts with a generic distinction of two types of narrative in discourses on migration (see section “Migration and narrative: Emic and etic perspectives”). The first type, stories of migration, present mobility from an inside (emic) perspective, as it includes various forms of self-expression, from conversational storytelling to artistic forms of communicating life stories, through images, audiovisual media, or literary representations (poetry, short stories, memoirs, novels). Ideally, stories of migration grant migrants full authority and control over their narrative. Pragmatically, though, narration is always subject to all sorts of constraints, and this is particularly obvious if fundamental issues like one’s status in asylum procedures are at stake. Thus, stories of migration always entail a struggle for narrative agency. ^2^

Narratives on migration, the second type, approach migration from an outside (etic) perspective. Examples are legal, political, economic, or scientific discourses. Such narratives can take the shape of master-narratives ( Lyotard, 1984 [1979]), mini-narrations based on metaphors ( Nünning & Sicks, 2012), conversational frames ( Goffman, 1986), or any kind of public discourse. ^3^ Migration studies is a particularly broad field, ranging from quantitative approaches in economics and the social sciences to literary criticism. What all scientific and scholarly narratives on migration have in common, however, is that, if they present any lived experience of migration at all, they do so primarily from an external perspective.

Stories of migration and narratives on migration form the extreme ends of a scale which accommodates various hybrid forms: migrant stories can be used to raise awareness, highlight the humanitarian catastrophe behind metaphorical expressions like “migration flows,” ^4^ and campaign for liberal migration policies. Such hybrid narratives can be differentiated with the help of functional criteria. The functional approach acknowledges that journalists, human rights groups, and NGOs supporting refugees and migrants at various stages of their journey (from transit and immigration to projects geared toward integration and inclusion) employ life stories for different reasons.

In order to account for this variety, this article employs the concept of vicarious storytelling. By this we mean the act of speaking on behalf of someone else which is typical of migrant advocacy and humanitarian narratives (see section “Hybrid forms: Migrant testimony and vicarious storytelling”). Vicarious storytelling also includes forms of narrative empowering, *i.e*., initiatives which provide opportunities for migrants to share their stories. Vicarious and empowering forms of storytelling seek to establish a humanitarian, pro-migration narrative. One should acknowledge, however, that stories of migration can also be used to discourage migrants from coming to Europe, to encourage return migration, or to amplify stereotypical representations of economic migration. ^5^ Such representations of migration don’t fall under the category of ‘vicarious storytelling.’

The Horizon 2020 project “Crises as OPPORTUNITIES: Towards a Level Telling Field on Migration and a New Narrative of Successful Integration,” funded by the European Union, ^6^ argues that migrant advocacy and humanitarian narratives are only a first step toward a fair debate on transnational mobility. In order to establish a new, fairer narrative on migration in European public arenas, we need to move beyond widespread forms of vicarious storytelling: rather than speaking for migrants, or about migrants, we should speak with–and listen to–them. In section “Toward a level telling field (LTF),” we therefore propose the notion of the ‘level telling field’ (LTF), a scalable concept based on the economic metaphor of a ‘level playing field’ ensuring fair conditions for competitors in a market. LTFs can be understood as playbooks and mechanisms guaranteeing a fair dialogue, on an equal footing, on various levels, from local events with migrants, aid workers, and stakeholders to public narratives on migration.

## Migration and narrative: Emic and etic perspectives

Narrative research in the last twenty years has emphasized the epistemological value, psychological use, and social function of storytelling as a means of knowledge production and worldmaking, self-fashioning and identity formation, or communication and community-building. ^7^ The act of narration, which involves event modeling and event management ( Sommer, 2023), can be viewed as a performance for the speaker’s intended audience. In this sense, storytelling as a process or activity is always embedded in a specific social context that informs the way a story is told. Social contexts also define a story’s tellability, *i.e.*, the “quality that makes stories inherently worth telling” ( Ryan, 2008 [2005], p. 589). ^8^ The voluntary decision to tell and share a story is a form of empowerment, turning storytellers into agents with a high degree of authority and control over their narrative. ^9^ This is particularly important in circumstances such as forced displacement and irregular migration that systematically deprive potential storytellers of narrative agency. ^10^ For this reason, narratives are of particular importance in the context of migration.

Stories of migration can take various forms, *e.g.*, informal oral storytelling among friends, family, acquaintances, aid workers, and volunteers, either in face to face communication or by phone; this is the dominant form of storytelling among migrants in transit who rely on this kind of information exchange in order to plan the next steps of their journey, make new contacts, share experiences, and find new motivation to carry on. ^11^ The harsh conditions in transit usually deny migrants the space, means, and peace of mind needed to produce verbal accounts for broader audiences. It is therefore not surprising that stories of migration tend to be retrospective accounts, written after the arrival in a new, safe country.

There are, of course, exceptions like the anthology *My Pen Won’t Break But Borders Will: Letters to the World from Moria* ( 2019–2020) by Parwana Amiri, a teenage refugee from Herat, Afghanistan. Her collection of 14 letters, partly autobiographical, partly fictionalized accounts of life in the infamous refugee camp, was published in 2019 on the Welcome 2 Lesvos blog run by a network of Greek and German activists. ^12^ The introduction describes Amiri’s working conditions: “These letters were written mostly at night by torchlight in the tent that Parwana shared with her eight-person family, in the olive grove. She always waited until everyone was asleep, so that she would have the peace of mind to write in the darkness with her torch.” (p. 5) Amiri’s project finds agency in a world without hope: “I am a girl in a tent” (p. 25), she notes, in a gesture of defiance and self-empowerment. This is worldmaking put into practice: Amiri’s writing begins to transform reality. In 2021, she was invited to participate in a hybrid panel, “School of Resistance,” an activist art project produced by Milo Rau for Schauspiel Köln (Cologne City Theater). ^13^

While cases like Amiri’s real-time stories are rare, retrospective stories of migration occur in different forms and contexts. Jad Turjman’s memoir *Wenn der Jasmin auswandert: Die Geschichte meiner Flucht* ( 2019) is the story of a Syrian refugee who managed to reach Austria, was granted political asylum, and started a promising career as a writer before falling to his death in a tragic mountaineering accident in 2022. *Little Brother: A Refugee’s Odyssey* ( 2019), a collaboration between Ibrahima Balde, a Guinean refugee, and Amets Arzallus Antia, an improvisational poet, is the retelling of Balde’s flight across the sea from Guinea to the Basque Country of Spain, where he shared his story with Arzallus Antia. The collection *Map of Hope and Sorrow: Stories of Refugees Trapped in Greece* ( 2022), edited by Helen Benedict and Eyad Awwadawnan, assembles stories of six refugees in detention who shared their remembered experiences of war, violence, and flight in interviews conducted by the editors. In a similar manner, Elika Ansari’s *The Five Stages of Moria* ( 2022) collects stories of refugees stuck in transit.

Blending autobiography and fiction, Dina Nayeri’s novel *Refuge* ( 2017) tells the story of Niloo, an Iranian migrant. Nayeri’s book *The Ungrateful Refugee: What Immigrants Never Tell You* ( 2019) is based on her own experiences and interviews with other migrants. Of course, migration novels are not restricted to autobiographical or autofictional forms but can make use of the full range of strategies and devices available to writers of fiction. Sefi Atta’s novel *The Bad Immigrant* ( 2021), for instance, uses a male protagonist, admitted through the Diversity Immigrant Visa Program, to represent a Nigerian immigrant’s experience of the USA, while Jason Donald’s *Dalila* ( 2017) focuses on a female protagonist from Kenya who, having survived rape in her home country, seeks asylum in Great Britain.

What these stories of migration have in common is that they are emic ( *i.e.,* insider) accounts of what it means to experience displacement, human trafficking, debt bondage, racism, or disillusionment. In contrast, political, legal, scientific, or economic narratives on migration approach migration from an etic ( *i.e.*, external) perspective. ^14^ They constitute what Doris Bachmann-Medick and Jens Kugele (2018, p. 3)–drawing on Erving Goffman’s (1986, p. 21) definition of frames as “schemata of interpretation”–have called “frames” of migration. Such frames, they argue, “constitute methodologically and epistemologically self-reflexive approaches to the complex field of migration”–approaches that are “effective in shaping the field of socio-political experience and behavior that directly impacts the lives of migrants” (p. 3). In other words, narratives on migration engender ideas that not only influence how we think and talk about migration, but also actively shape our understanding of different forms of mobility, as well as informing attitudes toward migrants and refugees ( De Coninck *et al.*, 2021).

The shared emic perspective doesn’t mean, however, that all narratives on migration are alike. From a theoretical perspective, it is clear that policy narratives, narrative advocacy and lobbying, economic narratives and the narratives generated by science and scholarship follow different discursive rules, address specific audiences, and fulfill a broad range of generic functions. Science and scholarship, for instance, follow guidelines for good research practice. As forms of second-order observation, in the terminology of Niklas Luhmann’s systems theory, they not only observe phenomena such as the role of storytelling in expressing and sharing experiences of mobility, but also address the principles and processes defining the narrative reconstruction of lived experience in abstract terms. Other narratives are evaluated against impact objectives or feasibility. The specific type and relevance of an emic perspective thus depends on a variety of pragmatic and epistemological factors.

Narratives on migration can frame mobility in a variety of ways. Nationalist narratives present migration as a threat to sovereignty, for instance, while economic narratives emphasize the financial burden of accepting large numbers of refugees. Legal narratives highlight the differences between regular and irregular migration, whereas humanitarian narratives insist on a human rights approach to migration. Most policy narratives consider migration as a problem that requires a solution, preferably one that–in the European context–is supported by all EU member states. Other narratives reframe migration as the only sustainable way to respond to the global problem of anthropogenic climate change ( Khanna, 2021; Vince, 2022). These environmentalist narratives, it seems, strive to form a counter-narrative ( Lueg & Lundholt, 2021) to dominant discourses, inviting us to re-evaluate our notions of migration and mobility.

Narratives on migration differ, however, not only with respect to their thematic focus, but also with respect to the ways in which–as well as the reasons for which–they address the topic of migration in the first place. Accounts like the annual World Migration Report, produced by the United Nation’s International Organization for Migration (IOM), for example, primarily fulfill an informative function, as they describe the status quo with the help of numbers and statistics. ^15^ Such narratives on migration are mainly descriptive, whereas legal narratives on migration qualify as normative, given that they establish rules and regulations which determine, for instance, under what circumstances migrants may be granted asylum in European countries. Political and humanitarian or human-rights based narratives, in contrast, qualify as ideological, albeit for different reasons: while the former are typically informed by election programs and party manifestos, the latter campaign for the rights of migrants by stressing the principles of the Universal Declaration of Human Rights in transnational contexts. Closely related to such humanitarian narratives are advocacy-oriented narratives on migration, which seek to empower migrants by speaking up for their rights ( *e.g.*, when lawyers support migrants in their attempt to seek asylum in a European Union member state).

## Hybrid forms: Migrant testimony and vicarious storytelling

However, the distinction between stories of migration and narratives on migration is not always as clear-cut as in these examples. Many hybrid scenarios embed migrant testimonials in narratives supporting humanitarian and journalistic efforts to raise awareness for refugees and migrants– *i.e.*, narratives *on* migration. Vicarious narratives of this kind can pursue various objectives such as documentation, perspective taking, or the evocation of empathy, and can do so with various levels of intensity. ^16^ Thus most vicarious storytelling will serve a documentary function, informing the audience about specific aspects of the lived experience of mobility. Not all vicarious narratives, however, will also stimulate perspective taking for migrants and refugees, ^17^ and even fewer will evoke empathy for their experiences of migration or displacement. Nevertheless, most vicarious narratives prompt readers and listeners to try on the perspective of migrants and refugees, enabling them to better understand migrant and refugee experiences. If perspective taking is successful, it can lead to the third objective of vicarious storytelling: the evocation of empathy. Vicarious narratives often resort to what Suzanne Keen (2007) calls “strategic narrative empathy”–that is, they seek to evoke empathy for a specific purpose, raising awareness of the situation of migrants by making their experiences more accessible to others. ^18^

But how exactly do vicarious narratives make use of migrant stories? How do they integrate migrant voices in larger narrative contexts to achieve the three main effects outlined above? Vicarious storytelling comes in different forms which are operative in different narrative environments ( *e.g.*, humanitarian, journalistic, scientific, or activist discourses). We distinguish four major types of vicarious storytelling in migration discourses: (1) case stories used in humanitarian campaigns, (2) documentary storytelling, (3) ambassadorial storytelling, and (4) allied storytelling. Case stories and documentary narratives merely use migrant testimony, often in anonymized form, to support claims, provide information, and illustrate facts. Ambassadorial narratives go a step further, focusing on specific individuals whose life story is featured through acts of reframing and retelling. Finally, allied narratives are specific cases of collaborative storytelling, usually in the context of literary or artistic projects where allies retell migrant stories.

The remaining part of this section examines each of these typical forms of vicarious storytelling in greater detail by referring to selective examples from NGO campaigns, experts in the field, journalists, and collaborative cultural projects. Our presentation of the individual types of vicarious storytelling focuses first on the framing and purposes of the chosen examples. We then proceed to discuss the ethical and social implications of vicarious narratives by addressing issues and concerns such as categorization and labeling, narrative agency, authority, and ownership, as well as storyteller’s positioning *vis-à-vis* normative discourses. This survey, however, is by no means exhaustive: more forms may well be identified in future research. Besides, various combinations of the typical forms distinguished here are possible: for example, the hybrid forms mediating between stories of migration and narratives on migration may themselves be hybrids, integrating various forms of vicarious storytelling. What is more, there are many forms that oscillate between the poles of stories *of* and narratives *on* migration; our typological differentiation seeks to structure the field in a descriptive rather than a normative manner.

### Case stories in humanitarian campaigns

Migrants’ stories are routinely employed in humanitarian campaigns by NGOs like Germany’s *Pro Asyl*, whose mission is to offer assistance to individuals, to document human rights violations, and to stand up for refugee protection. ^19^ Their recent flyer “Save the forgotten ones!”, a plea to rescue former local staff working for the German army in Afghanistan, now threatened with retribution by the Taliban, is an example of the use of migrant stories for advocacy and fundraising purposes. ^20^ Apart from the fundraising slogan (“Your contribution protects refugees!”), the flyer includes concise information on the flawed rescue mission in 2021, as well as on the current state of affairs in Afghanistan and on the German visa processing policy–a reference to promises made by the German government in 2021.

This information is backed by case stories, anonymized micro-biographies offering factual information– *e.g.*, professional qualification, role in the German administration, kind of assistance required–relating to individual Afghanis affected by the withdrawal of the German army. In one case, a reference is made to the desperate situation (“Hussein has fled, one of his brothers has been killed. Hussein has remained in hiding ever since, hoping desperately for a way out.” [authors' translation]), but the omission of further detail shows that an appeal to empathy, unlike in the ambassadorial storytelling discussed below, is not the point of this kind of case story. Nor are these stories designed to foreground migrant voices in public discourse; their purpose is to illustrate and support the political aims of *Pro Asyl* as an organization whose humanitarian narrative on migration is as simple as it is compelling: the individual matters.

### Documentary storytelling: Investigative journalism

Documentary storytelling is the main rhetorical strategy of investigative journalism. A prime example is Daniel Trilling’s work on the borders of Europe. Trilling, a British journalist writing for *The Guardian* and the *London Review of Books*, among others, is the author of *Lights in the Distance: Exile and Refuge at the Borders of Europe* ( 2018). In this book he investigates the strategies and mechanisms behind irregular migration to and within Europe. His main source of information is a pool of migrant stories gathered in interviews:

Clearly, the book relies on individual testimony. I wanted to present that in as open and honest a way as possible, which is why I have narrated our encounters in the first person. I also did it because I want you to see these people as I did, and not to let the harshest experiences overshadow the other aspects of their personalities. […] Some of my interviewees’ names have been changed, at their request. (p. 266)

Trilling’s work combines the retelling of the stories collected in conversation with background knowledge such as a description of the City of Calais, brief historical surveys to provide context, or information about, or gained from, aid volunteers and social workers, No Borders activists, local authorities, or the police. In contrast to ambassadorial narratives intended to evoke empathy in non-migrant audiences (see section “Ambassadorial storytelling: Humanitarian narratives in social media and journalism”), Trilling focuses on factual content rather than emotional impact: How do migrants move from A to B, what kinds of support networks can they rely on, how do they manage to overcome various obstacles while on the move, and what sources of information are available to them? Trilling’s exclusive concentration on story content is partly due to linguistic difficulties, as in the case of Zainab: “My Arabic was limited to a few nouns; her rudimentary English wasn’t enough to tell me her story in detail.” (p. 39) But although he uses translators to overcome the language barrier, in order “to hear Zainab’s story as she wanted to tell it” (p. 39), he retells her story mainly from a third-person perspective, with the interspersal of a few quotations.

The reason why emotional states, hopes, and aspirations do not play a key role here is programmatic: Trilling’s journalistic behind-the-scene approach to the realities of migration focuses less on individuals than on strategies, practices, networks, and relationships of trust between different groups of migrants and stakeholders. The goal of his conversations is neither to highlight the plight of the individual nor to compose fully-fledged biographical accounts of their lives, but to investigate “the effects of Europe’s border crisis on the people caught up in it” (p. xiii). His interlocutors are portrayed as representatives of a group, irregular migrants attempting to cross, or having crossed, the Channel to claim asylum in the UK.

### Ambassadorial storytelling: Humanitarian narratives in social media and journalism

While case stories and documentary storytelling mainly use migrant testimony for informative and illustrative purposes, ambassadorial storytelling draws on migrant life stories in order to call for humanitarian, social, or political action. A successful example of this form of vicarious storytelling is a TED Talk by Melissa Fleming, ^21^ former spokesperson of the United Nations High Commissioner for Refugees, which centers on the fate of Doaa Al Zamel, a Syrian refugee. Delivered in Thessaloniki, Greece, on May 23, 2015, the TED Talk presented Doaa’s fate for the first time to a public audience.

Analyzing Fleming’s biography of Doaa, ^22^ Ana Belén Martínez García (2021) shows how the author uses narrative strategies to evoke empathy, presenting Doaa as an individual (p. 216), emphasizing her role as an eyewitness (pp. 214–215), and granting readers an insight into her mind (p. 215). These strategies also structure the TED Talk, which places special emphasis on Doaa’s emotions, with Fleming showing pictures of her and her fiancé and using terms of endearment (“mum,” “dad”) when talking about her parents, which creates intimacy between Doaa and the audience. In this way Fleming establishes Doaa’s story as a narrative with a high degree of tellability: “Every day, I listen to harrowing stories of people fleeing for their lives across the dangerous borders and unfriendly seas. But there’s one story that keeps me awake at night, and it’s about Doaa.” (00:05–00:22)

The tellability of Doaa’s story is further enhanced by her presentation as “an extraordinary survivor.” In retelling Doaa’s journey from Egypt to Crete, Fleming combines two generic templates: an inverted rags-to-riches plot and the hero protagonist or the “victim-turned-survivor” theme ( Martínez García, 2021, p. 217). Fleming stresses that Doaa has lost everything in life, explaining why she lets her fiancé Bassam persuade her to leave Egypt and try to escape to Europe despite the fact that she cannot swim, and is therefore “terrified of the water” (01:45). The inverted rags-to-riches template enables the audience to understand why the dangerous journey to Europe is Doaa’s only chance of a good education and with it a better life. Fleming describes in detail the horrifying scenario when the boat carrying Doaa and Bassam eventually sinks, ^23^ stressing that, despite these traumatizing events, Doaa still manages to save two children from drowning–a transformation from victim to heroine that makes Doaa’s an exceptional fate among other refugee stories.

In addition to using strategies primarily designed to offer the audience access to Doaa’s experience, Fleming’s talk resorts to “metanarrative digressions” ( Martínez García, 2021, p. 215), *i.e.*, rhetorical questions which emphasize the problems that force people like Doaa to leave their home countries (“And let me take a pause in the story right here and ask the question: why do refugees like Doaa take these kinds of risks?” [06:43]). Fleming talks about causes of forced displacement ( *e.g.*, the civil war in Syria) and points to the severe problems and disastrous living conditions in refugee camps; while doing this, she shows pictures of destroyed cities as well as camps in the desert. Fleming presents these facts to criticize “the richer world” (08:28), which fails to make a sufficient effort to change the situation: “Why, the root question, is so little being done to stop the wars, the persecution and the poverty that is driving so many people to the shores of Europe?” (16:53) At the end of her presentation, she finally encourages her audience to “be inspired by what happened [to Doaa and other refugees] and take a stand for a world in which every life matters” (18:46). Fleming deploys Doaa’s story, then, as a means to stress the urgency of her call for humanitarian action. More specifically, her presentation draws on UNHCR’s central communication strategies, which she discloses in an interview with Martínez García: “(1) Lead the narrative, (2) Build empathy, (3) Drive action. So it’s: inform people, get them to care, and then give them something to do.” (Fleming qtd. in Martínez García, 2021, 219)

Another instance of vicarious storytelling can be taken to confirm this pattern, even though it is rooted in different media and discourse contexts, pursues a different purpose, and makes use of different narrative and rhetorical strategies. This second example of ambassadorial storytelling is a non-fiction book on forced displacement and migration by Melita H. Šunjić–also a former UNHCR spokeswoman. Published in 2021, *Die von Europa träumen: Wie Flucht und Migration ablaufen* ( *Those Who Dream of Europe: How Flight and Migration Take Shape*) presents nine migration stories. Unlike Fleming’s account which focuses exclusively on the fate of a single refugee, Šunjić’s text foregrounds diversity by focusing on nine migrants from Afghanistan, Eritrea, Iraq, Nigeria, Senegal, Somalia, and Syria. Šunjić uses a form of vicarious storytelling which employs abstraction and generalization rather than examples. The texts, she explains in the preface, neither refer to individuals, nor are they fiction. Rather, they are reconstructions on the basis of interviews with more than 2,000 asylum seekers from East and West Africa, the Near East, and Afghanistan, which the author has rearranged in order to ensure the anonymity of the respective interviewees (pp. 11–12).

What is the purpose of these stories? On the one hand, Šunjić wants to introduce voices of migrants to the European public debate on migration, which she considers overly Eurocentric (p. 11). On the other hand, she criticizes public migration discourse for having long tended to polarize societies, as it has been directed at creating affect rather than explaining the complex dynamics contributing to the debate (p. 12). Šunjić therefore complements the nine life stories with thematic chapters on key terms such as migrant, refugee, and asylum seeker (Ch. 10), and smuggling and human trafficking (Ch. 12), as well as with a discussion of central topics such as sea rescue (Ch. 13), integration and inclusion (Ch. 14), and European migration policy (Ch. 16). She resorts to ambassadorial storytelling in order to reframe and recontextualize migration discourses in a way that will inform and educate her readers.

### Allied storytelling: Collaborative literary and artistic work

Vicarious storytelling is also employed in the realm of literature, art, and other cultural productions. Story and essay collections like *The Refugees* ( 2017) and *The Displaced: Refugee Writers on Refugee Lives* ( 2018), both edited by Viet Thanh Nguyen, compile refugee narratives as retold by established contemporary authors and novelists. Theater productions like *Our Footsteps* (2019), a multilingual collaboration between artists, activists, and refugees in the Netherlands, ^24^ or the 2016 version of the *London Stories* by the Battersea Arts Centre in London, ^25^ have brought and re-enacted authentic migrant experiences on stage. Since 2021, Little Amal, the giant puppet of a ten-year-old Syrian refugee girl has been visiting and walking through more than ten countries across Europe, where numerous people have joined her in *The Walk*. ^26^ And the audiovisual installation *Carne y Arena*, created by Alejandro G. Iñárritu, enables audiences to experience what it’s like to step into the shoes of undocumented Mexican migrants trying to cross the border to the United States of America. ^27^

These projects seek to make experiences of flight and forced displacement accessible to a wider public. Indeed, authors and artists often lend their voice to migrants and refugees because these groups have little opportunity to participate in public debates on migration and integration. As a result, media representations are often distorted, failing to inform us about the lived experience of migration and displacement. In her review of the third volume of *Refugee Tales*, author Kamila Shamsie points out that “[w]e hear so many of the wrong words about refugees–ugly, limiting, unimaginative words–that it feels like a gift to find here so many of the right words which allow us to better understand the lives around us […].” ^28^ Shamsie’s comment stresses the potential of literature–and art for that matter–to serve as a counterbalance to biased media coverage of migration by introducing to public discourses the perspective of those who are directly affected by movements of migration. ^29^ A further strength of such collaborative projects is that they use multiperspectivity to emphasize diversity, bringing together migrants with different backgrounds and experiences in migration and displacement.

*Refugee Tales* ( 2016–2021), edited by David Herd and Anna Pincus, is an excellent example of what can be called *allied storytelling*. ^30^ The four volumes so far published grew out of the project “Refugee Tales: A Walk in Solidarity with Refugees, Asylum Seekers and Detainees,” a collaborative protest march of members of charities, activists, refugees, and authors to criticize and challenge the British immigration detention system. The first walk, organized in June 2015 by the Gatwick Detainees Welfare Group and Kent Refugee Help, took nine days, including several stops during which participants publicly performed two stories or “tales,” one being the life story of an asylum seeker or refugee, the other that of a lawyer or interpreter *etc.* working with those seeking asylum in the UK. Each story was the result of a close collaboration between established writers and the person whose story was presented, and all the narratives were subsequently published in the first volume of *Refugee Tales* ( Herd, 2016b, p. 133).

While the walks and events behind *Refugee Tales* are designed to address a local audience in the UK (the initial march, for instance, covered the route from Dover *via* Canterbury to Crawley), the literary product of these marches, the four volumes of *Refugee Tales*, addresses a wider readership. The purpose of this collaborative literary project is made explicit in the “Prologue” to the first volume, where Herd (2016a) explains that the book is “a declaration / […] Of solidarity” (p. v), that the act of walking serves “To make a spectacle of welcome” (p. vii), and that the stories collected in the volume are intended to create “a whole new language / Of travel and assembly and curiosity / And welcome” (p. viii). The stories in *Refugee Tales* represent an act of solidarity with migrants and refugees and the attempt to create a new welcome culture. To achieve this aim, they draw on the image of the walk as an active instrument calling for a new migration discourse that acknowledges the voices of those who are usually silenced in the British immigration system (p. ix): “And every step sets out a demand / And every demand is urgent / And what we call for / Is an end / To this inhuman discourse.” (p. x) ^31^

The experience of mobility constitutes a central element not only in *Refugee Tales*, but also in other projects that stand up for the rights of asylum seekers. The project Little Amal, the giant refugee puppet on the move, pursues a similar objective to that of *Refugee Tales*. Amir Nizar Zuabi, Artistic Director of *The Walk*, describes this as follows:

It is because the attention of the world is elsewhere right now that it is more important than ever to reignite the conversation about the refugee crisis and to change the narrative around it. Yes, refugees need food and blankets, but they also need dignity and a voice. The purpose of The Walk is to highlight the potential of the refugee, not just their dire circumstances. Little Amal is 3.5 metres tall because we want the world to grow big enough to greet her. We want her to inspire us to think big and to act bigger. ^32^

The quotation emphasizes several points already raised in previous examples. What distinguishes *The Walk* from these, however, is the endeavor to work toward a shared narrative *of* migration that migrants and welcoming societies worldwide can create *together*. In every city, town, or village in which she arrives, Little Amal, who stands for the sum of all refugee children in the world, is received with a special “Event of Welcome” such as a parade through the city center, a musical concert, or a dance performance. ^33^ Amal then walks across the place, accompanied by her local audience. By inviting spectators to literally share Little Amal’s experience of mobility through the physical re-enactment of her journey, the project sets the course for a new collective narrative on migration that appreciates notions of diversity, inclusion, and participation routinely excluded from migration discourse.

### Vicarious storytelling and social action

Our discussion of the typical forms of vicarious storytelling demonstrates that migration narratives can serve not only as framing strategies (as is the case with narratives *on* migration) or means of sense-making, identity formation, and empowerment (stories *of* migration), but also as a form of social action. The “practice-based ‘social interactional’ approach” to narrative, a framework of narrative analysis for the social sciences introduced by Ana De Fina and Alexandra Georgakopoulou ( 2008, 2015), allows us to expand our understanding of migration: How can narratives help to bring about societal change–for instance through measures that seek to establish a welcome culture for migrants–and thus lay the foundations for a more diverse and inclusive society? Narratives are shaped by communicative contexts, yet they also “create new contexts by mobilizing and articulating fresh understandings of the world, by altering power relations between peoples, [and] by constituting new practices” ( De Fina & Georgakopoulou, 2015, p. 3). Conceptualizing narrative as social action in this way foregrounds the ethical implications of vicarious storytelling, in particular issues of categorization, ownership, and positioning.

Although vicarious storytelling pursues a good cause, it also incurs the risk of categorizing individuals. Life stories of migrants and refugees are highly complex, and their state of migrancy or refugeedom only constitutes one of many dimensions of their identity. Public discourses, however, often give rise to simplified forms of vicarious storytelling such as case stories that reduce the complexity of migrants’ life stories to specific labels such as the labor migrant, refugee, or asylum seeker. A similar tendency can be observed in forms of ambassadorial storytelling which produce shorter narrative content: Fleming’s TED Talk, for instance, deploys specific generic templates to present Doaa as a victim who turns into a heroine. Other forms of vicarious storytelling, in contrast, actively work against such practices of labeling, as is the case in Šunjić’s non-fiction book and the *Refugee Tales*, which strive to create multiperspectivity by granting readers insight into a wide spectrum of migrant and refugee experiences.

The issue of ownership raises important questions: Who has the right to tell or retell a story and how can acts of telling and retelling influence the authority of both owners and tellers of stories? According to Amy Shuman (2015, p. 38), “[much] is at stake in contests and questions about who owns a story and who is entitled to tell it or hear it.” This is especially true in migration debates, which are informed by various discourses that uphold uneven power structures diminishing the rights of migrants and refugees: To what ends should narratives on migration draw on the life stories of migrants, without granting the latter narrative agency and authority? Who has the right to speak for migrants, to claim their vicarious voice, if the latter are not able to share their stories themselves? Under what circumstances does advocacy lead to the empowerment of migrants, and in what situations does it have the opposite effect, increasing their marginalized status within society?

When fundraising campaigns make use of brief case stories, migrants are usually framed as victims in order to create empathy and increase the willingness to donate money. Such frames, however, contribute to victimization. From an ethical perspective, every retelling of migrant stories “complicate[s] (and undermine[s]) the unstated rule that the person who suffered or experienced the event has the right to tell it” (p. 41). As Fleming explains in the “Author’s Note” at the end of *A Hope More Powerful than the Sea*, Doaa wanted her story to be heard, but would not have been able to share it with the public herself, for it was too traumatic ( 2017a, pp. 269–270). She consequently depended on Fleming as a mediator to help her render her experiences tellable and communicate them to a wider public. In cases of collaborative storytelling projects such as the *Refugee Tales* or *The Walk* of Little Amal, by contrast, the question of ownership and narrative authority is transferred to a collective that works together to construct a new narrative which is shared by all participants. As the examples of vicarious storytelling discussed in the previous sections serve to illustrate, each retelling creates new discourse contexts in which ownership and narrative authority “can be refigured, reclaimed, and/or contested” ( Shuman, 2015, p. 41).

Migrant advocacy generally pursues a good cause, yet one must bear in mind that all forms of vicarious storytelling carry a degree of responsibility. As Shuman reminds us, the practice of lending a vicarious voice to groups which are underrepresented in public discourse does not automatically increase fairness in discussions of migration or other related issues: “The act of narrating does not necessarily change the conditions of marginalization that underlie access to speaking for oneself or that assign some events to public and others to hidden status. On the contrary, giving voice to the voiceless can just as often reproduce the power relations underlying a group’s or speaker’s status.” (p. 41) In order to avoid such untoward effects, vicarious narratives often resort to strategies of positioning, which clarify their standpoint within the current debate on migration. ^34^

## Toward a Level Telling Field (LTF)

Advocating the rights of migrants, vicarious narratives are the backbone of humanitarian discourses of migration. Unlike mainstream narratives on migration, which typically frame migration as a crisis or a threat, strategic retelling foregrounds the plight of migrants, as well as their political claims and human rights. By feeding back these perspectives into a public debate that might otherwise ignore what happens in real life and in real time, vicarious storytelling challenges Eurocentric discourses on migration which normalize extreme responses ( *e.g.*, fences, illegal pushbacks, zero solidarity policies). What is more, vicarious narratives can operate against simplifying discourse practices that reduce refugees and migrants to suppliants and petitioners, highlighting the personality and rights of individuals. In this sense, vicarious narratives are instrumental in empowering refugees and migrants.

Despite the best efforts of vicarious storytelling, however, the European border crisis will not be solved by only speaking on behalf of others. The current discursive climate, which is characterized by racist stereotypes, labeling and blaming on the one hand, and mistrust among European partners, lack of solidarity, and nationalist selfishness on the other, reveals a series of systemic problems. These include a lack of a common vision among European member states, a constant shift toward more radical positions, and a *laissez-faire* attitude with respect to the inhumane treatment of new arrivals in overcrowded and ill-equipped detention camps or during illegal pushbacks.

Systemic problems require systemic solutions. One way to work toward a fair narrative *on* migration is to rethink the principles of conversation and to advocate mutual recognition among all parties involved, as well as an ethics of listening to experiment with innovative forms of social action. Fairness is the key issue here. In sports and economics, the metaphor of the level playing field indicates endeavors to create equal opportunities for all players and competitors and promote fair play. Global trade relies on level playing fields to ensure that “all countries and firms compete on an equal footing to offer consumers everywhere the widest possible choice and the best value for money.” ^35^ In political theory and media research, this translates into deliberative democracy and various forms of participation, as well as calls for the representation of dissenting voices and marginalized perspectives in mainstream media.

From the perspective of transdisciplinary narrative research, the specific challenges we face when dealing with discursive practices – rather than with rules (sports), policies (trade), or political participation – can be emphasized by the modified metaphor of the ‘level *telling* field’ which emphasizes communicative practices. By analogy with fair play or fair trade, level telling fields (LTFs) advocate fair competition between narratives, concepts, and ideas. In migration discourses, this involves a healthier balance between stories of migration and narratives on migration.

LTFs are playbooks and mechanisms for an open, constructive, and productive debate–the cornerstone of a democratic, pluralist, secular society. They are best viewed as commitments by all participants in a debate to adopt a shared set of premises, to agree on principles and rules, and to define processes and procedures for conducting debates and documenting results. LTF premises include: a) a commitment to a democratic worldview grounded in human rights and a human development paradigm ( Nussbaum, 2010); b) adhering to commonly accepted standards for evaluating claims, opinions, and arguments; and c) sincerity, *i.e*., a serious commitment to debate as a democratic means of opinion-building and decision-making. An LTF approach to migration insists that all participants in a debate subscribe to these premises and principles, and defines a set of procedures for ensuring a fair conversation. Just as leveling the metaphorical playing field, however, leveling the telling field is also a complex task. As German philosopher Jürgen Habermas (2022), a pioneer of deliberative democracy, has argued recently, we need to rethink representation as well as forms and functions of the public sphere in the face of decentralizing digital technologies.

The LTF approach, then, introduces the concept of fairness in a debate currently dominated by exclusionist arguments and nationalist policies to create obstacles to mobility. Leveling the telling field involves protecting narrative ownership, while at the same time encouraging European audiences to listen more carefully. The concept thus offers a new framework for rethinking migration discourses not as a battle of frames, but as a fair dialogue and open-ended conversation on the great challenges of our times. Treating refugees and migrants not as suppliants and petitioners, but as experts with first-hand experience of the many global crises that affect us, directly or indirectly, is an opportunity Europe cannot afford to miss.

## Conclusion

This article has approached the narrative dynamics of migration from a typological perspective in a three-step argument. We first distinguished two major types of narrative representing either an insider’s (emic) or an outsider’s (etic) view of migration. The former type, we argued, includes stories of migration told by refugees and migrants themselves, *i.e.*, narratives grounded in lived experience; the latter refers to the economic, political, legal or academic narratives on migration which dominate both migration policies and the public perception of refugees and migrants.

Proceeding from this distinction, the article laid particular emphasis on a hybrid, in-between type of vicarious storytelling on behalf of refugees and migrants. Such humanitarian narratives *on* migration integrate stories *of* migration in order to emphasize their authenticity, generate empathy in audiences, and put migrant perspectives on the agenda. To further differentiate these various purposes, we introduced four subtypes of vicarious narrative: (1) case stories, (2) documentary storytelling, (3) ambassadorial storytelling, and (4) allied storytelling.

Although speaking on behalf of migrants is a legitimate strategy to advance a humanitarian narrative, vicarious storytelling raises ethical questions, as it ascribes to refugees and migrants a largely passive role that fails to do justice to the communicative potential of storytelling as a form of empowerment and recognition. In this article, we therefore propose a new approach to narrative communication, based on the notions (derived from sports) of fair play and level playing fields. Both of these concepts are routinely used in economic contexts concerned with fair competition. Our new concept, the Level Telling Field (LTF), helps us reconsider the ways we talk about migration by providing us with a new set of discourse rules that enable us to work toward a fairer and more inclusive debate on the topic.

Although migration studies is “a broad and diverse research field” ( Scholten *et al.*, 2022, p. 4) with a high degree of interdisciplinarity ( De Haas *et al.*, 2020, p. 44; Scholten *et al.*, 2022, p. 3), conceptual transfer between economics, sociology, and political science on the one hand, and literary and cultural studies on the other is still in its infancy. Yet the humanities, we would argue, have considerable potential in cross-disciplinary research on migration. The *Introduction to Migration Studies* ( 2022), edited by Peter Scholten, is a case in point. This comprehensive cross-disciplinary overview of the field is an excellent resource in many ways, yet it fails to include approaches like narrative theory, postcolonial and subaltern studies, critical race theory and diaspora studies, intercultural studies, or gender and queer studies. Challenging existing power structures from various angles, these approaches are, however, vital for a more inclusive migration debate, and should therefore be integrated into any cross-disciplinary migration research. Recent studies have also highlighted the relevance of the humanities for a “politics of mobility” ( Cresswell, 2006; Cresswell, 2010) and, more broadly, the field of mobility studies ( Aguiar *et al.,* 2019; Merriman & Pearce, 2018).

With respect to narrative research, further exploration of the forms of vicarious storytelling is clearly an important task. The survey in section “Hybrid forms: Migrant testimony and vicarious storytelling” above is a first attempt at describing the uses, functions, and effects of stories for migrant advocacy in a systematic manner. This preliminary typology could be expanded into a fully-fledged taxonomy which would benefit not only the study of narrative and migration but also current research on life writing, as well as supporting recent approaches to human rights narratives and activist storytelling ( Hopkinson & Marsh, 2020; Martínez García, 2020). A theoretical discussion of the narrative dynamics of vicarious storytelling would, moreover, help to develop the analytical paradigm of cross-disciplinary narrative research.

Given that vicarious storytelling is not the only type of hybrid narrative that falls within the paradigm of ‘stories *of*’ and ‘narratives *on*,’ further research could also investigate the different ways in which narratives on migration make use of migrant testimony and stories of migration. Since our paper has focused primarily on migrant advocacy, we have largely ignored how discourses on migration can–and sometimes do–instrumentalize migrant stories for ill-intentioned purposes. Such hybrid cases would also provide a fruitful basis for discussing the discursive power structures which privilege narratives *on* migration over stories *of* migration, thus strengthening the social hierarchies that prevail between migrants and local citizens. Another important research question concerns the ways and circumstances in which migrants can influence narratives on migration themselves– *i.e.*, without the mediation of vicarious storytellers–when engaging in politics or migration research.

Finally, we would like to emphasize that the LTF approach is not limited to migration. It seeks to overcome gridlock scenarios, challenges partisan and tribal politics, and addresses widespread feelings of anger, frustration, and anxiety ( Mishra, 2017; Shafak, 2020) at the closing of public space in times of uncertainty. LTF playbooks and mechanisms are a way of examining the shifting boundaries between public and private communication and other consequences of digital technologies, such as growing pressure on the old functions and business models of legacy media, and the rise of new platforms for curated information. The LTF paradigm may also develop new diagnostic tools for evaluating narrative dynamics in the public sphere ( Habermas, 1992; Habermas, 2022), and for detecting and countering threats to democratic systems of checks and balances ( Ziblatt & Levitsky, 2018).

## Ethics and consent

Ethical approval and consent were not required.

## Data Availability

No data are associated with this article.

## Footnotes

^1^ A recent example of this rhetorical strategy is the argument presented in Douglas Murray’s *The Strange Death of Europe: Immigration, Identity, Islam* ( 2018). Referring to the refugee crisis in 2015/2016, Murray points out that German authorities “recorded an additional 680,000 arrivals into their country in 2016 alone” (p. 257), with serious consequences: “Such continuing mass immigration, high birth rates among immigrants and low birth rates among native Europeans ensured that the changes underway would only accelerate in years ahead.” (p. 257) Murray here alludes to–and fuels–the Islamophobic conspiracy narrative of the great population exchange, also known as the so-called ‘Replacement Theory’ ( Thiem, 2020, p. 301), which blames feminist indoctrination for falling birthrates among white women. It is no surprise, then, that he defines “mass immigration” as “the replacement of large parts of the European populations by other people” ( Murray, 2018, p. 3) and claims that “the civilization we know as Europe is in the process of committing suicide” (p. 1).

^2^ For the sake of clarity, we wish to point out that the term *story* is used in a generic sense and does not refer to the classical narratological distinction between ‘story’ ( *histoire* in Gérard Genette’s sense) and ‘discourse.’

^3^ For a discussion of the popular uses of the terms *story* and *narrative*, see Mäkelä & Björninen, 2023, pp. 21–22.

^4^ Other metaphors that are frequently used in public debates on migration include container metaphors or metaphors describing migrants as commodities or objects (see, e.g., Arcivamiciene & Hamza Baglama, 2018; Charteris-Black, 2006, pp. 570–575; Petersson and Kainz, 2017, pp. 53–55).

^5^ In addition to this, voices of migrants can also be instrumentalized by politicians and political institutions for legitimizing anti-immigration policies ( Björninen *et al*., 2020, pp. 442–444) or promoting migration management and return migration measures ( De Jong & Dannecker, 2017).

^6^ For more information on OPPORTUNITIES, see the project’s website: https://www.opportunitiesproject.eu/ (accessed November 7, 2022). The project has received funding from the European Union’s Horizon 2020 research and innovation programme under Grant Agreement no. 101004945.

^7^ See, *e.g.*, Bamberg, 2013, Nünning *et al.*, 2010, and Müller-Funk, 2008 [2002].

^8^ As sociolinguist Neil R. Norrick (2005) has observed, tellability is limited by two boundaries: stories must be sufficiently interesting to find an audience, and they must not touch on issues which are regarded a taboo in a given context.

^9^ Agency is a controversial concept whose philosophical implications have been discussed by Hannah Arendt (2008 [1958], p. 184): Even though human beings may think of themselves as agents, the idea of agency, authority, and control is, in the greater scheme of things, an illusion. In the context of ecocriticism, the idea of human agency has more recently been challenged by the concept of nonhuman agency; for a critical discussion see Caracciolo (2022, p. 140), who points out that storytelling is a “quintessentially human practice.”

^10^ For a comprehensive discussion of storytelling in asylum application procedures, see Bohmer and Shuman, 2008.

^11^ See Trilling, 2018; his journalistic account of migrant experiences offers numerous examples.

^12^ See http://lesvos.w2eu.net/ (accessed November 7, 2022).

^13^ See https://www.schauspiel.koeln/en/schedule/a-z/school-of-resistance/ (accessed November 7, 2022).

^14^ From a narratological angle, David Herman (2009) has redefined the anthropological distinction between an “etic” and an “emic” perspective as follows: “[A] question for any account of the basic elements of narrative is whether those elements are in fact oriented to as basic by participants engaged in storytelling practices (= emic), or whether the elements are instead part of a system for analysis imposed on the data from without (= etic).” (p. 3)

^15^ The World Migration Report has been published since 2000 on a regular basis. All reports can be read and downloaded at: https://worldmigrationreport.iom.int/what-we-do/all-world-migration-reports (accessed August 15, 2024).

^16^ Our understanding of vicarious narrative is inspired by Mari Hatavara and Jarmila Mildorf ( 2017a, 2017b), who, drawing on Norrick’s (2013) understanding of “narratives of vicarious experience,” define vicarious storytelling as “the use of stories about the experience of someone other than the narrator” (2017a, p. 394).

^17^ Cognitive approaches to narrative “[distinguish] two ways of adopting other’s perspective: the ‘imagine-self’ perspective and the ‘imagine-other’ perspective” ( Nünning, 2014, p. 182). While the former tends to induce egocentric behavior (p. 237), involving the act of imagining how we would think and feel if we were in the other’s place (p. 182), the latter can elicit altruistic and selfless behavior (p. 237), as it entails imagining how another person is thinking and feeling (p. 182). The use of migrant stories in narratives on migration can promote both types of perspective taking, depending on the various purposes of vicarious storytelling.

^18^ Keen (2007) distinguishes three types of narrative empathy–bounded, ambassadorial, and broadcast–each of which is directed at a different audience (p. 142). Bounded empathy addresses an in-group; “stemming from experiences of mutuality,” it invites the audience “to feeling with familiar others” (p. 142). Ambassadorial empathy includes “chosen others,” seeking to “[cultivate] their empathy for the in-group, often to a specific end” (p. 142). Broadcast empathy encourages everyone “to feel with members of a group,” as it stresses “common vulnerabilities and hopes” (p. 142).

^19^ See https://www.proasyl.de/en/ (accessed November 7, 2022).

^20^ See https://www.proasyl.de/wp-content/uploads/Posterfl_web_Afgh.pdf (accessed November 7, 2022).

^21^ See https://www.ted.com/talks/melissa_fleming_a_boat_carrying_500_refugees_sunk_at_sea_the_story_of_two_survivors (accessed November 7, 2022).

^22^ After her TED Talk turned out a huge success, Fleming approached a literary agent, asking whether Doaa’s story could be turned into a book ( Fleming, 2017b, p. 268). Two years later, in 2017, Fleming published Doaa’s biography *A Hope More Powerful than the Sea* in two editions, one for adult readers ( Fleming, 2017b) and one for young readers ( Fleming, 2017a). The latter offers additional material such as a map of the Mediterranean and the Levant, a glossary of Arabic terms, a timeline of important events in Doaa’s life, and an interview with photographer Brandon Stanton (Humans of New York).

^23^ More specifically, sentences like “Doaa was holding on to the side of the boat as it sank, and watched in horror as a small child was cut to pieces by the propeller” (04:54); “Doaa and Bassam watched as men in the distance took their life vests off and sank into the water” (06:00); or “he released himself into the water and Doaa watched as the love of her life drowned before her eyes” (09:16) are aimed at prompting readers to vividly picture the scene and to do so with empathy.

^24^ See https://ourfootsteps.nl/ (accessed November 7, 2022).

^25^ See https://www.theguardian.com/stage/theatreblog/2016/nov/12/london-stories-made-by-migrants-battersea-arts-centre-london (accessed November 7, 2022).

^26^ See https://www.walkwithamal.org/ (accessed November 7, 2022).

^27^ See https://phi.ca/en/carne-y-arena/ (accessed November 7, 2022).

^28^ See https://www.refugeetales.org/books (accessed November 7, 2022).

^29^ In fact, recent studies have started to highlight the value not only of literature, but also of theatre, film, and other visual arts to fight common stereotypes prevailing in media representations of migrants and refugees (see, *e.g.*, the contributions in Bayrakdar and Burgoyne, 2022, González Ortega and Martínez García, 2022, and Meerzon *et al.*, 2020).

^30^ The term *allied storytelling* is inspired by the concept of “white allies” in the context of critical race theory (see Muwanga forthcoming).

^31^ That the “Prologue” is written in verse is no coincidence, but a tribute to the medieval poet Geoffrey Chaucer, whose *Canterbury Tales* are the literary template on which the “tales” in the volumes are modeled. The intertextual reference ought to be taken not only as a literary artifice, but also as an indication that migration and mobility are far from being new. On the contrary, they go back to the Middle Ages and even earlier.

^32^ See https://www.walkwithamal.org/ (accessed November 7, 2022).

^33^ See https://www.walkwithamal.org/about-us/little-amal/ (accessed November 7, 2022).

^34^ For a detailed survey of the history of positioning theory and a discussion of its relevance for narrative research, see Depperman, 2015. Inspired by social psychology, positioning approaches in narrative analysis investigate the nexus between processes of identity formation and narrative in various contexts ( Bamberg, 1997; Bamberg, 2004); recent work by Samuli Björninen, Mari Hatavara, and Maria Mäkelä (2020) discusses narrative positioning in political storytelling, including possible “dangers of narrative” ( Mäkelä *et al.*, 2021) as well as harmful effects of storytelling (see also Freeman & Meretoja, 2023; Meretoja, 2017; Meretoja, 2018; Nünning & Nünning, 2017; Meretoja & Davis, 2018; and Presser, 2018). These approaches prove particularly beneficial when it comes to analyzing how storytellers position themselves *vis-à-vis* value-oriented and normative discourses as they engage in practices of vicarious storytelling.

^35^ See https://www.oecd.org/trade/topics/levelling-the-playing-field/ (accessed November 7, 2022); for a detailed discussion of the economic implications of level playing fields, see Messkoub, 2022.
