# Supplementary material for: Beyond Vicarious Storytelling: How Level Telling Fields Could Help Create a Fair Narrative on Migration
Source: Open Res Eur. 2024 Aug 20;3:10. Originally published 2023 Jan 16. [Version 2] doi: 10.12688/openreseurope.15434.2 (PMC11420614; doi:10.12688/openreseurope.15434.2)
Supplement: Peer review history

# Reviewer report: Reviewer response for version 2

**DOI:** 10.21956/openreseurope.19889.r43364

**Published:** 2024-09-23

**Authors:** Stefan Rother

**Affiliations:** 1 University of Freiburg, Freiburg, Germany; 2 University of Hamburg, Hamburg, Germany

In my review, I had pointed out two issues wich I recommended for further reflection. Both issues have been adressed in an appropriate manner and the revisions in response to the second review have further enhanced the paper.

 Therefore, I can gladly approve the indexing of the article without  reservations - congratulations to the authors for this relevant contribution to the field !

Is the argument persuasive and supported by appropriate evidence?

Partly

Is the work clearly and cogently presented?

Yes

Is the topic of the essay discussed accurately in the context of the current literature?

Yes

Does the essay contribute to the cultural, historical, social understanding of the field?

Yes

Reviewer Expertise:

Migration, social movements, human rights, migrant civil society, development.

I confirm that I have read this submission and believe that I have an appropriate level of expertise to confirm that it is of an acceptable scientific standard.

# Reviewer report: Reviewer response for version 1

**DOI:** 10.21956/openreseurope.16685.r30705

**Published:** 2023-02-27

**Authors:** Stefan Rother

**Affiliations:** 1 University of Freiburg, Freiburg, Germany; 2 University of Hamburg, Hamburg, Germany

This is a very well-written and useful article, providing a comprehensive overview on the various forms of (benign) migration storytelling and narratives as well as a critical analysis of the literature.

 I would like to address two issues, one rather minor, related to the typology, one somewhat larger, related to the title of the paper. 

1. I found the "four major types of vicarious storytelling in migration discourses"  identified by the authors convincing. 
  One aspect - leading to a potentially fifth type of storytelling - that the authors might reflect upon is the underlying assumption that the telling of migrant stories is mostly made with benign intentions. ( *Note: I have written this before reading the second-to-last paragraph where the issue is briefly mentioned for the first time; for the clarity of the paper, this focus should be included at a much earlier stage; additionally, I would encourage the authors to consider including this aspect as part of their typology, even if there is no space to elaborate on it in-depth*). There are the very obvious examples of anti-migrant actors, painting migrants and refugees in a negative light. But there are also more complex intentions as well.
  There are campaigns by countries of destination such as Sweden, Germany etc. aiming to discourage potential migrants in the countries of origin by painting a grim picture of dangers, failures etc. More generally, the examples for storytelling in the paper could be more explicitly discussed from a country/community of origin perspective, such as the stories of returned migrants/refugees (I am aware that this is one of the - laudable - aspects of the Opportunities Project, but existing storytelling in the origin should also be reflected upon). 
  There are also seemingly benign campaigns which nonetheless have received criticism for using individual storytelling to frame migration in a very specific - if not limited -  context, such as the #Iamamigrant campaign by IOM (not IMO as in the text).
  See: de Jong, Sara and Dannecker, Petra (2017). Managing Migration with Stories? The IOM “i am a migrant” Campaign. *Journal für Entwicklungspolitik*, XXXIII(1) pp. 75–101.
  This issue is somewhat touched upon in the section on "the risk of categorizing individuals" but could be expanded. 
2. One larger issue - and the reason why I chose "partly" in the question on  "supported by appropriate evidence?" - is related to the title which I found somewhat misleading. "How level telling fields help create a fair narrative on migration" - based on this title I would have expected a detailed analysis of such level telling fields to form the core of the paper. Instead, we get a critical analysis of the state of research - which is fine - followed by an outlook for an ongoing  research program. It is great to hear that "Storytelling projects [are] organized by NGOs in Romania, Italy, Austria, Belgium, France, Portugal, Ghana, Senegal, and Mauritania" but we learn nothing about them.
  The easiest way to address this would probably be a renaming of the paper - at the very least add a "could" before "help create a fair narrative on migration", although I would suggest a more significant change.
  The current title would be much more appropriate for a presentation of the findings and lessons learned during the project; I look very much forward to leaning more about them once they become available.

Is the argument persuasive and supported by appropriate evidence?

Partly

Is the work clearly and cogently presented?

Yes

Is the topic of the essay discussed accurately in the context of the current literature?

Yes

Does the essay contribute to the cultural, historical, social understanding of the field?

Yes

Reviewer Expertise:

Migration, social movements, human rights, migrant civil society, development.

I confirm that I have read this submission and believe that I have an appropriate level of expertise to confirm that it is of an acceptable scientific standard, however I have significant reservations, as outlined above.

# Response

**Published:** 2024-08-07

**Authors:** Carolin Gebauer

**Affiliations:** University of Wuppertal, Germany

We wish to thank Stefan Rother for this valuable feedback. The two issues he raises in his review are certainly worth considering and have helped us to refine our main argument.

 We agree that narratives which draw on migrant stories aren’t always benign and may, for instance, be used to discourage and deter potential migrants. We have added a few such scenarios in the Introduction of our article, and we agree that this is an important issue for future research on narrative representations of migrants and migration. Our typology, however, explicitly foregrounds the act of speaking on behalf of others (hence, the terminological decision to call that kind of storytelling *vicarious*).

 The second issue raised by Rother relates to the conception of the Level Telling Field. We have tried our best to address this concern by elaborating on the guiding metaphor of the level playing field, from which it is derived, and we have also changed the title of the article, as suggested, and have reduced references to the project in order to avoid raising false expectations. Having said this, the metaphorical Level Telling Field is deliberately conceived not as a well-defined category but a programmatic concept designed to initiate conversations on fairness in controversial debates (including, but not limited to, migration discourses).

 Finally, it was our conscious decision to leave out vicarious storytelling in countries of origin. The situation in Senegal and Ghana, to name but two examples, introduces new complexities which exceed the scope of this paper: the dominance of social media and the specific challenges of forced migration, internal migration, and return migration all play a role, as do German attempts to recruit skilled labor in West Africa.

# Reviewer report: Reviewer response for version 1

**DOI:** 10.21956/openreseurope.16685.r30661

**Published:** 2023-02-09

**Authors:** Mari Hatavara, Matti Hyvärinen

**Affiliations:** 1 Finnish Literature, Tampere University, Tampere, Finland; 2 Tampere University, Tampere, Finland

Friendly and vicarious?

 A review comment on Carolin Gebauer and Roy Sommer

 Carolin Gebauer and Roy Sommer address a topical, highly relevant, and scholarly inspiring issue in their article. A great number of migrants travel to Europe, and more would come if allowed. Whether we speak about migrants, refugees, or asylum seekers, the stories on migration matter politically, socially, and humanely. Gebauer and Sommer not only focus on narratives in contest (Phelan 2008) but especially address the crucial issue of story ownership (Shuman 2005) and the phenomenon of vicarious storytelling (Norrick 2013) in the context of migration. Their contribution belongs to and enriches the emerging study of narrative realities (Gubrium and Holstein 2007), the study of socially and politically functioning significant narratives. In this review, we want to attest to the general approach of the authors and their main results. At the same time, we want to raise some critical, theoretical, and conceptual issues about the essay for the purposes of facilitating discussion. While we fully agree that the article is a strong scholarly contribution in its current form, we also encourage the authors to consider if some of our questions could help in developing their ideas further.

 Throughout their essay, Gebauer and Sommer discuss narrative control (cf. Gubrium and Holstein 2007, 109-122) and different rights to tell. As they say, “migration narratives always entail a struggle for narrative agency”. The authors pursue a more balanced and fair visibility between external (political, economic, scientific) and migrants’ internal stories accounting their experiences of migration. In a continuum between these two end points the authors locate various cases of vicarious narratives where migrants’ stories are instrumentally used by generally well-meaning NGOs and other organizations. The authors distinguish four types of such vicarious storytelling: “(1) case stories used in humanitarian campaigns, (2) documentary storytelling, (3) ambassadorial storytelling, and (4) allied storytelling”. As often with scholarly distinctions, it is easier to agree with the critique the authors present towards the first cases than to fully believe in the optimistic or utopian end of the continuum they suggest.

 The authors advocate for “a new humanitarian narrative on migration, one that moves beyond vicarious storytelling to enable refugees and migrants to speak for themselves, thus protecting narrative ownership and encouraging European audiences to listen more carefully” (p. 11). This is an excellent objective. However, does it repudiate vicarious stories a bit too categorically? Before condemning all vicarious stories, we should possibly remember that vicarious stories can express closeness (old couples telling their stories), help (patients with dementia or speaking problems), or solidarity (social movements sharing their stories). Adriana Cavarero (2000, 55-65) seems even to privilege vicarious life-stories in comparison with self-told stories. We would take a more moderate position and suggest that vicarious stories can still play a useful role in many contexts. This is something that Gebauer and Sommer also seem to suggest in their complimenting treatment of allied storytelling - also (at least partly) a form of vicarious storytelling.

**Emic and etic**

 The question of vicarious stories is connected to another conceptual distinction the authors make in their essay. They write, quite boldly, “what all scientific and scholarly narratives on migration have in common, however, is that, if they present any lived experience of migration at all, they do so primarily from an external perspective” (p. 3). Even with the recognition of the modifier “primarily”, this claim is remarkably strong, at least while presented without discussion on possible options. This categorical evaluation is supported by two concepts borrowed from anthropology – *emic* and *etic*. Anthropologists use these terms to describe *their own theoretical work* and its categories in relation to phenomena they encounter “on the field”. In the etic approach, scholars bring in their own theoretical (psychological, evolutionary, whatever) distinctions and concepts and use them in the analysis of their findings. In the emic approach, the scholar wants to study the distinctions and structures of meaning-making that are relevant for and used by those who are part of the studied culture. What is more, anthropologists usually agree that both these analytic moves are needed, and an entirely emic approach may be impossible.

 Gebauer and Sommer, however, transfer these terms from the analytic practice and research orientation to characterize the materials they study, that is their data, suggesting that only stories told by migrants themselves can be emic, and “(i)n contrast, political, legal, scientific, or economic narratives on migration approach migration from an etic […] perspective” (p. 5). Paradoxically, this claim leaves anthropologists and other scholars only the external, etic perspective, which is what Gebauer and Sommer wanted to overcome by suggesting the heuristic distinction between emic and etic in the first place. What is more, we should strongly resist the temptation to position scholarly attempts at understanding migration into the same category with dominant economic or political stories and allow more space for ourselves as researchers.

 Similar confusion can be detected in the use of the concept “voice”. In criticizing, for perfectly good reasons, what the authors call “case stories in humanitarian campaigns”, they also write “Nor are these stories designed to give migrants a voice; their purpose is to illustrate and support the political aims…” (p. 6). Later, in describing their favourite alternative of “allied storytelling”, Gebauer and Sommer write: “Indeed, authors and artists often lend their voice to migrants…” (p. 8). In terms of Richard Walsh, voice is understood here only as an *instance* and not as an *idiom* connected with subjectivity and social groups, or *interpellation*, containing the layers of other people’s voices (Walsh 2007, 86-102). In early qualitative social research, it was usual and well-meaning but often self-complacent to argue that the researcher was “giving a voice” to the marginalized groups. In the case of migrants, for example, this manner of approaching is arguably misleading. Since the immigrants already have their voices, the assumed benevolent act of giving or lending a voice seems to be just another form of patronizing. The problem rather is at the other end of the communication, the missing or indifferent audiences and listeners.

 Those in power are able to give the right to speak, write, and vote, and try to listen and understand. In the Bakhtinian tradition, voice is not the pure origin of subjectivity, but always also socially and historically layered. (See Rautajoki & Hyvärinen, 2021.) Here vicarious storytelling could also be an opportunity to adopt and adapt elements of migrants’ voices into our scholarly voices. Rather than trying to give voice, we might and should position the migrants as the speakers. 

**Level Habermasian field?**

 As we already mentioned, it is always easier to criticize the problematic practices (which the article does very convincingly) than to provide functioning alternatives. Gebauer and Sommer suggest the useful concept of “allied storytelling”, showing how activists, artists, and migrants have worked together in various projects with the purpose of generating new stories. As the case of Viet Thanh Nguyen indicates, it might sometimes take decades for the migrants to tell independently and competently stories that are seriously recognized within the dominant culture.

 The most optimistic, if not utopian, objective in the paper is the *Level Telling Field*, an idea the authors draw from economic discourse about fair trade. It is, however, not easy to fully understand the analogy from firms competing over the consumers' attention to migrants’ possibilities to tell their stories. In the case of trade, economic theory and the basic idea of the EU support the levelling of the terms of trade. In the case of migration, the divergence of opinions and political interests cuts deeper, and there are prominent political agents who rather distribute the external stories in order to block access to Europe. In the post-Brexit world, the fields of telling tend not to be very level, and Jürgen Habermas’ idea of “domination free discussion” appears more than utopian. What the project by Gebauer and Sommer has generated, however, seems to be a space for mutual and diverse storytelling. The problem, possibly, is not only about level telling fields or migrants’ voices to be heard. As the powerful example of Viet Thanh Nguyen seems to indicate, the migrants can also profit from richer command of the narrative resources of the dominant culture. When having these resources, he was more competent in writing his compelling counter-narratives.

 Still, it is not entirely clear how extensive, restricted, or fleeting the possible level fields could possibly be.

 Even within small, egalitarian, and temporary projects huge differences exist in regard to social and cultural capital. The terrain outside of these projects would obviously be even rockier and steeper (cf. Phelan 2008, 122). Rather than searching an utopian state of a level field, we would suggest something like “safe, experimental narrative environments”, which could possibly conjoint the (older) European cultural and narrative resources with the practice of “treating refugees and migrants not as suppliants and petitioners, but as experts with first-hand experience of the many global crises…” (Gebauer & Sommer, 11). 

**Story and narrative**

 As narratologists, we are also concerned about the distinction made in the abstract: “Starting with a generic distinction between what we call *stories* of migration (various forms of self-expression granting migrants full authority and control over their narrative) and *narratives* of migration (external perspectives, e.g. academic, economic, political, and legal approaches, where lived experience doesn’t matter…” (emphasis added). The authors, however, never define the concepts of story and narrative. Later, the authors connect “stories of migration” with the *emic *perspective, and “narratives on migration” with the *etic*, external perspective. We recognize that research always struggles with giving fitting names to new phenomena and to coin necessary distinctions. We are also fully aware that many if not most social scientists consider story and narrative as synonyms and prefer then to talk about stories (while the term, indeed, is more emic than narrative). Some theorists have even tried to make the distinction evaluative by seeing stories nice and people-friendly, and narratives as dangerously ideological (e.g. Boje 2001).

 Narratology makes the distinction between story as the contents (happening, persons) of the told and narrative as also involving the act of telling and the properties of the discourse. Particularly in a research where storytelling is - rightly, to our mind - considered interconnected to agency, it would be helpful to maintain this distinction between story as the content only and narrative as also always including the situation and act of telling. Narratives are, indeed, social acts, where the stories told are only part of what needs to be considered in interpretation (cf. Björninen et al. 2020).  Stories, if taken apart from their tellers and contexts of telling, have the capacity of being the most ideological and harmful messages from migrants’ perspective. Therefore, we think that both stories and narratives can be benevolent or ideologically harmful. The distinction between the two could greatly help in analyzing the many positions between the speaker and those portrayed in the story, crucial particularly to vicarious storytelling.

 -----

 To conclude, we want to thank Gebauer and Sommer for their important and innovative contribution. We have suggested some conceptual clarifications above, and hope they help and inspire the researchers for further research and for cultivating their model. Perhaps some clarification on the basic conceptual background and new suggestions could also help facilitate the usability of the results Gebauer and Sommer provide.

 References:

 Björninen, S., Hatavara, M, Mäkelä, M. (2020). Narrative as Social Action: A Narratological Approach to Story, Discourse and Positioning in Political Storytelling. *International Journal of Social Research Methodology*. 23(4): 437-449. DOI: 10.1080/13645579.2020.1721971

 Boje, D. M. (2001). *Narrative Methods for Organizational and Communication Research*. London & Thousand Oaks & Delhi: Sage.

 Cavarero, A. (2000). *Relating narratives: Storytelling and Selfhood* (P. A. Kottman, Trans.). London and New York: Routledge

 Gubrium, J. F., & Holstein, J. A. (2009). *Analyzing Narrative Reality*. Los Angeles, CA, London: Sage.

 Norrick, N. (2013). Narratives of Vicarious Experience in Conversation. *Language in Society,* 42(4): 385–406.

 Phelan, J. (2008). Narratives in Contest; or, Another Twist in the Narrative Turn. *PMLA, *123(1): 166-175.

 Rautajoki, H. & Hyvärinen, M. (2021). Aspects of voice in the use of positioning in polyphonic storytelling: Ventriloquial moves within a biographical interview. *Journal of Interdisciplinary Voice Studies* 6(2): 55-73. DOI: 10.1386/jvs_00037_1

 Schuman, A. (2015). Story Ownership and Entitlement In A. De Fina & A. Georgakopoulou (Eds.), *The Handbook of Narrative Analysis* (pp. 38--56). Chichester: Wiley Blackwell.

Is the argument persuasive and supported by appropriate evidence?

Partly

Is the work clearly and cogently presented?

Yes

Is the topic of the essay discussed accurately in the context of the current literature?

Yes

Does the essay contribute to the cultural, historical, social understanding of the field?

Yes

Reviewer Expertise:

Narrative studies

We confirm that we have read this submission and believe that we have an appropriate level of expertise to confirm that it is of an acceptable scientific standard.

## References

[1] : Narrative as social action: a narratological approach to story, discourse and positioning in political storytelling. *International Journal of Social Research Methodology* .2020;23(4) : 10.1080/13645579.2020.1721971 437-449 10.1080/13645579.2020.1721971

[2] : Narrative Methods for Organizational and Communication Research. *London* .2001;

[3] : Relating narratives: Storytelling and Selfhood (P. A. Kottman, Trans.). *London and New York: Routledge* .2000;

[4] : Analyzing Narrative Reality. *Los Angeles, CA, London: Sage* .2009;

[5] : Narratives of vicarious experience in conversation. *Language in Society* .2013;42(4) : 10.1017/S0047404513000444 385-406 10.1017/S0047404513000444

[6] : Narratives in Contest; or, Another Twist in the Narrative Turn. *PMLA/Publications of the Modern Language Association of America* .2008;123(1) : 10.1632/pmla.2008.123.1.166 166-175 10.1632/pmla.2008.123.1.166

[7] : Aspects of voice in the use of positioning in polyphonic storytelling: Ventriloquial moves within a biographical interview. *Journal of Interdisciplinary Voice Studies* .2021;6(2) :55-73 Reference source

[8] : Story Ownership and Entitlement In A. De Fina and A. Georgakopoulou (Eds.), The Handbook of Narrative Analysis (pp. 38-56). *Chichester: Wiley Blackwell* .2015;

# Response

**Published:** 2024-08-07

**Authors:** Carolin Gebauer

**Affiliations:** University of Wuppertal, Germany

We highly appreciate Mari Hatavara and Matti Hyvärinen’s insightful response to our article. The theoretical and conceptual issues they raise have encouraged us to clarify some aspects of our approach to migration narratives.

 Following Hatavara and Hyvärinen’s suggestions, we have expanded our discussion of migration narratives so that it now offers a more nuanced perspective on both vicarious storytelling and narratives on migration. While we still address the ethical dilemma of the act of speaking on behalf of others, we now explicitly emphasize the positive effects of vicarious storytelling. Besides, we specify the difference between political and media discourses on the one hand and scientific and scholarly accounts on the other, clarifying that the latter constitute, in the terminology of Luhmann’s systems theory, forms of second-order observation. We moreover inserted a footnote clarifying that we use the term *story* in a (broad) generic sense, not in its narratological meaning ( *histoire* in Gérard Genette’s understanding) which rests on the distinction, and opposition, between story and discourse. We are grateful to the reviewers for pointing out the potential for misunderstanding here, and hope it has now been resolved.

 In addition to these theoretical points, we wish to thank Hatavara and Hyvärinen for suggesting that the imbalance between narratives on migration and stories of migration problematized in our article might result not so much from a lack of migrant voices in public discourses, but rather from “missing or indifferent audiences and listeners.” This is certainly a complex issue, which requires reference to specific contexts; although we maintain that migrant voices are under-represented, it is a valid objection that getting heard requires the attention of an audience. However, our typology proceeds from the observation that vicarious storytelling is such a dominant practice in migration discourses that it needs to be addressed in more detail. We have also responded to the reviewers’ comments on our core concept, the Level Telling Field, by explaining how the idea of fair trade inspired the metaphorical concept of the level telling field as a guiding vision to consider fairness in migration debates.
